# Autophagy as a Possible Underlying Mechanism of Nanomaterial Toxicity

**DOI:** 10.3390/nano4030548

**Published:** 2014-07-08

**Authors:** Vanessa Cohignac, Marion Julie Landry, Jorge Boczkowski, Sophie Lanone

**Affiliations:** 1 Institut National de la Santé et de la Recherche Biomédicale (INSERM), U955, Equipe 4, Créteil 94000, France; E-Mails: vanessa.cohignac@inserm.fr (V.C.); marion.landry@inserm.fr (M.L.); jorge.boczkowski@inserm.fr (J.B.); 2 Université Paris Est, Faculté de Médecine, Créteil 94000, France

**Keywords:** nanomaterials, oxidative stress, inflammation, autophagy, lysosomes

## Abstract

The rapid development of nanotechnologies is raising safety concerns because of the potential effects of engineered nanomaterials on human health, particularly at the respiratory level. Since the last decades, many *in vivo* studies have been interested in the pulmonary effects of different classes of nanomaterials. It has been shown that some of them can induce toxic effects, essentially depending on their physico-chemical characteristics, but other studies did not identify such effects. Inflammation and oxidative stress are currently the two main mechanisms described to explain the observed toxicity. However, the exact underlying mechanism(s) still remain(s) unknown and autophagy could represent an interesting candidate. Autophagy is a physiological process in which cytoplasmic components are digested via a lysosomal pathway. It has been shown that autophagy is involved in the pathogenesis and the progression of human diseases, and is able to modulate the oxidative stress and pro-inflammatory responses. A growing amount of literature suggests that a link between nanomaterial toxicity and autophagy impairment could exist. In this review, we will first summarize what is known about the respiratory effects of nanomaterials and we will then discuss the possible involvement of autophagy in this toxicity. This review should help understand why autophagy impairment could be taken as a promising candidate to fully understand nanomaterials toxicity.

## 1. Introduction

Nanotechnologies represent a major technological advance of this last century. Their objects are engineered nanomaterials (at least one dimension in the nanometer range) which possess astonishing physical and chemical properties. The list of actual applications and uses for nanomaterials is already substantial, and will certainly become exponential in the future [1]. In parallel, questions raise regarding the potential (human and environmental) toxicity of these nanomaterials, particularly at the respiratory level. These questions are justified by the knowledge of the toxic effects of micrometric particles from atmospheric pollution on human health, and the fear to get an amplification of these effects because of the (nano) size of the materials blamed.

In this review, we will go through the actual knowledge available in the literature regarding the pulmonary toxicity of nanomaterials, and will discuss on autophagy as a novel mechanism possibly underlying this toxicity.

## 2. Pulmonary Toxicity of Nanomaterials

Among the nanomaterials family, carbon nanotubes (CNT) and metal oxide nanoparticles (NP) have received the most attention, because of their present and foreseen large production and use in the industry. The following paragraphs are dedicated to describe the lung remodeling manifestations that are currently described in the literature as respiratory consequences to pulmonary exposure to these nanomaterials. Indeed, although it has been described that nanomaterials could enter the blood stream, translocate to other organs [2,3,4], and induce extra-pulmonary effects, we focused on the respiratory system as it is the better explored as of today. However, in this review we will focus on the respiratory effects as consequences of pulmonary exposure to these nanomaterials. The underlying biological mechanisms proposed at the moment in the literature and the physico-chemical determinants of these effects will be exposed to get a comprehensive overview and understanding of the respiratory toxicity of nanomaterials.

### 2.1. Lung Remodeling Manifestations

The existing literature on pulmonary health effects of nanomaterials related to respiratory exposure is relatively new [5]. It consists of experiments performed with mice or rats mainly exposed by intratracheal or oro-pharyngeal administration, but also (although more recently) by aerosol. The majority of these studies have mainly focused on two pulmonary manifestations: the formation of granuloma and/or the development of pulmonary fibrosis. Such lung remodeling effects can occur after pulmonary exposure to various types of nanomaterials, namely CNT, iron oxide NP, titanium dioxide (TiO_2_) NP, cadmium-doped silica NP, quantum dots, *etc.*, [6,7,8,9,10,11,12,13]. Granuloma formation, consistent with foreign body response [14,15,16], has been described within hours after the initial nanomaterial administration [12,13] and persist for weeks/months after [10,11,13]. Fibrosis on the other hand generally takes a few weeks to develop and remains persistent even after a few months [13,16,17]. This histopathological modification can occur either within granulomas or as diffuse interstitial and septal fibrosis, distal to granulomas.

Beside these well-described lung remodeling effects, nanomaterials administration has been in some cases also associated to the development of emphysema-like alterations, as shown after administration of TiO_2_ or Fe_2_O_3_ NP in mice [18,19]. Moreover, the development of mesothelioma, a malignant cancer of the mesothelium most commonly caused in response to asbestos exposure [20], has been reported after exposure to CNT or carbon nanofibers (CNF) [21,22,23]. Mesothelioma formation was observed in the visceral mesothelium, accompanied by hyperplastic proliferative lesions, inflammatory cell infiltration and inflammation-induced fibrotic lesions of pleural tissue in the lungs of rats exposed to MWCNT [24].

More recently, studies have been performed to address the issue of the pulmonary effects of nanomaterials in the context of preexistent respiratory diseases such as asthma or infection. For example, it has been shown that nickel NP cause exaggerated lung and airway remodeling in T-bet^−/−^ mice, the lack of this transcription factor being implicated in allergic airway inflammation that characterizes asthma [25]. Similarly, exposure to CNT can enhance the susceptibility of mice to develop airway fibrosis in murine models of asthma [26,27]. The same exacerbated fibrotic response has also been described when CNT were administrated concomitantly to Gram positive (*Listeria monocytogenes*) and Gram negative (*E. coli* lipopolysaccharide) bacteria; in both cases, CNT exposure in combination with bacterial infection was able to induce an increased airway fibrosis as compared to bacterial infection alone [28,29]. Finally, TiO_2_ NP can either aggravate or protect from airway inflammation and hyperresponsiveness, depending on the dose and timing of NP administration [30,31]. Similar protection has been described for Ag NP, as their pulmonary administration suppresses mucus hyper secretion in asthmatic mice [32]. Overall, the pulmonary effects of nanomaterials in susceptible individuals are currently far from understood. Moreover, it is important to underline that all nanomaterials do not systematically induce lung remodeling (in terms of presence/absence and intensity), depending on various factors (both intrinsic and/or extrinsic to the nanomaterials themselves). This clearly deserves further studies to fully understand the overall (respiratory) toxicity of nanomaterials.

### 2.2. Underlying Biological Mechanisms

Several biological mechanisms have been described as potentially underlying nanomaterials toxicity. The generation of an oxidative stress and/or the induction of an inflammatory response are the most often evoked (and usually linked), but the literature now also discusses the implication of genotoxicity, as well as the importance of the protein corona surrounding the nanomaterials. These mechanisms are discussed below.

#### 2.2.1. Oxidative Stress

Oxidative stress is defined as the imbalance between the production of reactive oxygen species (ROS) and antioxidant defenses, where the pro-oxidant forces exceed the antioxidant forces. Oxidative stress has been proposed to be a common mechanism in nanomaterials pathogenicity [33,34]. Several footprints of oxidative stress have been detected in the broncho-alveolar lavage fluid (BALF) and/or in the lung of mice or rats exposed to various nanomaterials, such as CNT [16,35], TiO_2_ NP [36] or iron oxides [37]—see [38] for review. For example, this includes the pulmonary expression of Heme Oxygenase-1 (HO-1), a marker of oxidative insult, the presence of lipid peroxidation products such as 4-HydroxyNonenal or 8-isoprostane, and the depletion of glutathione in BALF. These *in vivo* observations were also confirmed *in vitro* in various cell types (either primary cells or cell lines) exposed to nanomaterials [39,40].

The contribution of oxidative stress to nanomaterials toxicity is further exemplified by the fact that oxidant generation is important in developing a toxic response after nanomaterials exposure. Indeed, Shvedova and co-workers demonstrated that mice maintained on a vitamin E deficient diet showed an increased oxidative stress following SWCNT exposure [41]. This was associated with a higher sensitivity to SWCNT-induced acute inflammation: increased number of inflammatory cells and pro-inflammatory cytokine production (tumor necrosis factor α (TNFα), interleukin 6 (IL6)) and pro-fibrotic response. Similar results were obtained with positively charged Si-core NP [42]. Interestingly, a vitamin E-rich diet protects asthmatic rats from the exacerbation observed in response to CNT [43]. Finally, it has been shown that mice lacking NADPH oxidase, a pro-oxidant enzyme that generates superoxide radicals, exhibited a decreased superoxide anion production by neutrophils and an enhanced acute inflammatory response after SWCNT exposure by pharyngeal aspiration, together with a decreased profibrotic response [44]. All together, these results demonstrate the role of oxidative stress as a mechanism underlying nanomaterials toxicity.

#### 2.2.2. Inflammation

The induction of an inflammatory response has been described in numerous *in vivo* studies, after exposure of rats or mice to various nanomaterials [45,46,47] see [48] for review. This inflammation is characterized by an early onset, with the recruitment of neutrophils and macrophages in the BALF a few hours only after nanomaterials exposure [47]. This tissue infiltration is usually diminished a few weeks after the initial exposure, although it can persist up to one month [10,49]. The recruitment of inflammatory cells is accompanied by the release of pro-inflammatory cytokines, such as IL1β, IL6, monocyte chemoattractant protein 1 (MCP1), macrophage inflammatory protein 2 (MIP2) or TNFα in the BALF as well as in the lung tissue [46,49,50]. *In vitro* studies further identified at least macrophages [51], fibroblasts [52], epithelial [53] and mesothelial cells [54], as potent inflammatory cytokine producers in response to nanomaterials.

#### 2.2.3. Genotoxicity

Due to the size of the nanomaterials, the probability of their internalization into the cells and their interaction with the intracellular environment such as the nucleus is very high. These interactions can damage the genetic material and lead to genotoxic responses characterized by DNA damage and mutations that compromise the successful functioning of the cells and therefore their survival. Genotoxicity can happen because of a direct interaction of nanomaterials with the genetic material of the cells or it can be indirect genotoxicity due to the generation of oxidative stress that in turn will induce oxidative damage to the genetic material [55,56].

Recently, the genotoxic potential of various nanomaterials was reported [57]; several studies demonstrated the existence of DNA damage, chromosomal aberrations or micronucleus induction after exposure to nanomaterials, including metal-based NP and CNT, *in vitro* as well as *in vivo* [58,59,60,61]. For example, An and co-workers demonstrated that the interaction of DNA with carbon NP resulted in DNA binding and aggregation both *in vitro* and *in vivo* in a dose-dependent manner [62]. Another study showed that metal-based NP can tightly bind to DNA nucleobases, but also to Watson-Crick base-pairs AT and GC [63]. Besides a direct interaction with DNA, nanomaterials are also able to bind to the active site of proteins implied in DNA “care” leading to their conformational or structural changes, or resulting in a competitive inhibition of the enzyme [64]. Such an event has been described for C60 fullerene that interacts with the human DNA topoisomerase II α, leading to the inhibition of the enzyme activity [65]. Moreover, this nanomaterial may also interact with several proteins involved in the DNA mismatch repair pathway [66].

As discussed before, various nanomaterials have been shown to induce oxidative stress. It is known that ROS can directly attack DNA and generate modified DNA bases. Indeed, nanomaterials such as TiO_2_ NP can induce genotoxicity and impair DNA repair activity in cells, via their production of ROS [67], although it is not always true [68,69]. Moreover, pre-treatment with the free radical scavenger N-acetyl-l-cystein (NAC) leads to the inhibition of CNT or zinc oxide NP-induced genotoxicity [70,71].

#### 2.2.4. Interaction with the Protein Corona

When in contact with a biological environment, nanomaterials are rapidly coated with biomolecules that may modify their properties and the way in which they interact with cells [72]. This surface-bound coating is a dynamic mixture of proteins and lipids, called the protein corona. It has been argued that the interaction unit with the cell is not the nanomaterial by itself but the nanomaterial together with its corona of proteins issued from serum and other body fluids [73,74]. Careful studies have revealed that the composition of this corona is dynamic [75], reflects the size, shape, and surface properties of the nanomaterials, and finally, that it is a major determinant of the localization and subsequent effects of nanomaterials *in vivo* [76]. The two main consequences of the formation of this protein corona can be (i) the modification of the nanomaterials (surface) characteristics and further reactivity [77]; and (ii) the modification of the proteins that interact with the nanomaterial, possibility leading to their altered structural conformation and functionality. Both of these events can be important to nanomaterials toxicity [78,79].

Modification of nanomaterials secondary to their interaction with the protein corona has been exemplified for CNT, which could bind pulmonary surfactant proteins A and D, leading to a susceptibility for lung infection and emphysema in mice [80]. Interestingly, such nanomaterial modification by their proteic surroundings can also enhance their biocompatibility [77] or enable the protein-modified CNT to be non-toxic or less toxic than the pristine ones [79]. Importantly, the formation of a protein corona is dependent on the physico-chemical characteristics of the nanomaterials, and particularly their surface properties. Indeed, studying six different polystyrene NP varying in size and surface chemistries, Lundqvist and colleagues demonstrated that both size and surface properties play a very significant role in determining the protein coronas on particles of identical initial chemistry [76]. The same is true for CNT, which interaction with serum proteins seems to be depending on the intrinsic physico-chemical properties of the CNT [81].

Another important issue of this protein corona formation is that it can induce modifications in the absorbed proteins. To address this issue, Banerjee and co-workers investigated the conformational and functional properties of a large multimeric protein, α-crystallin, absorbed on silver NP surface [82]. The authors demonstrated that the chaperone function and the refolding capacity of the protein, which is primarily governed by the α-crystallin domain, were lost to a significant extent when adsorbed onto silver NP surface, because of the selective alkylation of two cystein residues at the α-crystallin domain. Nonetheless, the secondary structure of α-crystallin was mostly retained. Another evidence of such protein modification by nanomaterial interaction has been given by Chen and colleagues who showed that when CNT were bound to α-chymotrypsin, the complex could inhibit the enzymatic activity [83]. Finally, a study measured the extent and kinetics of internalization of acid-coated quantum dots, with and without adsorbed native and modified human serum albumin by HeLa cells [84]. Pronounced variations were observed, indicating that even small physico-chemical changes of the protein corona may affect biological responses.

It is important to understand that a nanomaterial entering the pulmonary system may pass through the mucosal layer and enter into the blood stream. At the cellular level, the nanomaterial may moreover be phagocytized and taken to the endosomes that ultimately fuse with lysosomes. Each of these steps represents unique environments, with specific characteristics, that could cause nanomaterial modifications, and therefore lead to the modification of the protein corona formation [85]. Nanomaterials entered in the body have thus to be considered as evolving systems, that are far from being understood yet.

### 2.3. Physico-Chemical Determinants

As stated before, all nanomaterials do not systematically induce identical effects (in terms of toxicity, lung remodeling, *etc.*), and the same is true for the biological mechanisms underlying these effects. According to the literature, the (nano)size of nanomaterials is not the only physico-chemical characteristic that plays a critical role in their toxicity [48]. Indeed, among these important characteristics (but the list remains open) are also the chemical composition, shape, crystalline structure, surface chemistry and charge. Importantly, such characteristics, alone or because of interplays among them, will condition the behavior of the nanomaterials, particularly their ability to form aggregates/agglomerates, which will also be a determinant of nanomaterials toxicity.

As it has been discussed in the literature, nanomaterials, whether they are carbon-based, TiO_2_, iron NP or other nanomaterials, can induce different biological effects depending on their chemical composition [86]. Moreover, nanomaterials of the same chemical composition, can also exhibit different behaviors [87,88], depending for example on the crystalline structure [52,89], the shape [52], or the number of walls for CNT [88]. For these latter nanomaterials, the length also seems to be important. Indeed, over the past decade, several studies demonstrated that long CNT were more pathogenic than the short ones. However, a more recent investigation observed a higher inflammogenic reactivity for short MWCNT as compared to long MWCNT, most likely due not only to the length reduction but also because of the accompanying surface modifications induced by the length reduction process [90].

Because of synthesis methods, nanomaterials can vary in regards to their remaining contaminants. Potential contaminants of CNT for example include residual metal catalysts (such as iron) that are used during their manufacturing process. These transition metals can induce toxicity by the production of ROS, and Kagan and co-workers demonstrated that, in conditions without cells, SWCNT with 26% iron had a greater potential to produce free radicals than their iron-depleted counterparts (0.23%) [91]. In cellular conditions, the iron-rich SWCNT were able to induce more oxidative stress than the iron-depleted SWCNT on murine macrophages. Moreover, metal chelators were able to reduce the toxicity observed on keratinocytes exposed to iron rich SWCNT (30%) [92], further demonstrating the importance of residual metal contaminants in nanomaterials toxicity. Interestingly, it was recently shown that iron NP can get detached from SWCNT (initially containing 25% iron) in murine macrophages, possibly via a pH-dependent mechanism [93]; the blockage of lysosomal acidification prevented this detachment and protected the cells against SWCNT toxicity. All these data suggest that the remaining contaminants play an important role in nanomaterials toxicity, and that they should be taken into account while assessing nanomaterial toxicity.

Another very important physico-chemical characteristic that needs to be considered is that regarding the surface of nanomaterials. A study from Tabet *et al*. [10] demonstrated that MWCNT-induced cytotoxicity, oxidative stress and inflammation were increased by acid-based and decreased by polystyrene-based polymer coating both *in vitro* in murine macrophages and *in vivo* in lung of mice monitored for 6 months. Similarly, another study observed that polyacrylate-coated TiO_2_ NP exhibited less cytotoxicity and induced no DNA damage on lung fibroblasts compared to their non-coated counterparts [94]. These findings suggest that surface chemistry of nanomaterials has the ability to modify their behavior and subsequent toxicity.

The aggregation or agglomeration state of nanomaterials also has to be taken into account when looking at nanomaterials effects. Nanomaterials tend to agglomerate (“weak” chemical bonds) and/or aggregate (“strong” chemical bonds) [95]. These unique and larger structures—that can reach the microscale—constituted of nanomaterial aggregates and/or agglomerates are more complex and more difficult to characterize. As it changes properties such as the surface area, it may modify the deposition into the lung, and therefore nanomaterial effects. Kreyling and co-workers showed that the translocation, from the lung of rats to their blood and other organs, is higher for agglomerates composed of 20 nm primary diameter iridium NP as compared to 80 nm ones [2]. In contrast, Noel and colleagues demonstrated a similar pattern between agglomerates of different sizes; after their inhalation, TiO_2_ agglomerates of 30 and 185 nm (primary diameter of NP) showed a similar lung deposition and both agglomerates resulted in comparable adverse effects in rats [36].

From all these studies, it appears that if data from the literature largely support the hypothesis that the physico-chemical characteristics of nanomaterials are important determinants of their toxicity, it is currently very difficult to clearly identify one characteristic of higher importance than another; one has to consider the role of the intricacy of these characteristics rather than the importance of one single characteristic alone. Such remark also reflects the difficulty to modify one characteristic at a time, and therefore to address its exact role in the nanomaterial’s toxicity.

As illustrated in this short overview of nanomaterials toxicity, it appears that pulmonary exposure to nanomaterials can lead to lung remodeling, with a probable important contribution of the physico-chemical characteristics of the nanomaterials. Although several biological mechanisms have been suggested to underlie these effects, they cannot, however, explain all nanomaterials toxicity. Recently, autophagy has emerged as a potential contributor of nanomaterials toxicity. This will be discussed in the following paragraphs.

## 3. Autophagy

Autophagy, derived from the Greek roots for « self-eating », is a general term standing for a cellular catabolic process in which cellular components, including organelles and macromolecules, are delivered to the lysosomes for degradation. Three types of autophagy have been described: microautophagy, chaperone-mediated autophagy and macroautophagy. Microautophagy involves the invagination of the lysosomal membrane, which leads to the sequestration and degradation of cytosolic components into the lysosome. Chaperone-mediated autophagy involves the selective translocation of altered proteins across the lysosomal membrane through chaperone proteins, which allows their recognition and unfolding, and, through the membrane receptor LAMP2a (lysosomal-associated membrane protein 2a), allows the translocation of the protein into lysosomes.

Macroautophagy, hereafter referred as autophagy, is characterized by the formation of a double membrane vesicle called autophagosome, which sequesters the cytoplasmic material to be degraded. Autophagosome then fuses with a lysosome to form an autolysosome in which the lysosomal enzymes will degrade the cargo. The resulting degradation products are recycled to maintain nutrient and energy homeostasis.

In most cell types, autophagy occurs at basal rate to maintain normal cellular homeostasis by eliminating misfolded proteins and damaged organelles. However, this process can be induced under stress conditions, such as metabolic stress (amino acid or growth factors deficiency), hypoxia or reticulum stress, to allow cell survival. As such, autophagy has been shown to play a key role in diverse pathologies, such as cancer, neurodegenerative, inflammatory and pulmonary diseases [96,97]. The involvement of autophagy in these pathologies can be associated to its role in the modulation of oxidative stress and inflammatory responses. As inflammation and oxidative stress are the most widely described mechanisms underlying nanomaterial-mediated toxicity, a growing amount of studies suggest that autophagy could be a potential new mechanism explaining, at least in part, the toxicity of nanomaterials. In this part of the review, we will describe the autophagy process in detail and we will briefly present evidences of its involvement in diverse pathologies.

### 3.1. Autophagy Machinery

**Figure 1:**
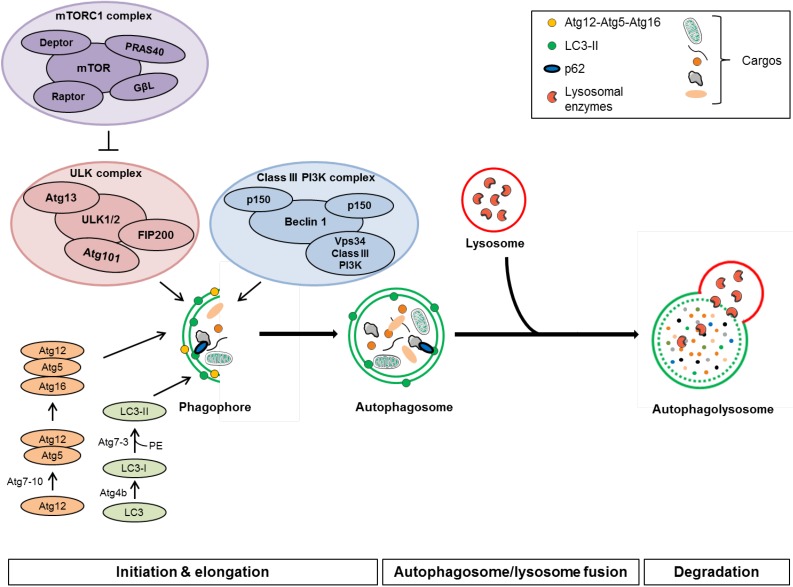
Scheme of the autophagic pathway.

The autophagy machinery can be divided into several steps: initiation, autophagosome formation, and autophagosome-lysosome fusion followed by the degradation and recycling of the cargo (see Figure 1). More than 30 genes are involved in the regulation of this process.

#### 3.1.1. Initiation

During the initiation step, an isolation membrane, called phagophore, is formed around the cargo. The exact origin of this membrane is not yet completely identified. Indeed, there may be multiple sources: the phagophore could be generated *de novo* from preexisting intracellular precursor proteins but it could also be derived from cellular structures such as endoplasmic reticulum [98,99], outer membrane mitochondria [100], Golgi [101] or plasma membrane [102]. The initiation step of autophagy involves two major macromolecular complexes: the mTORC1 (mammalian target of rapamycin complex 1) and the class III PI3K complex (class III phosphatidylinositol-3-kinase).

The mTORC1 complex is composed of the protein kinase mTOR, Raptor (regulatory associated protein of mTOR), GβL (mammalian G protein β-subunit-like protein), Deptor (DEP domain-containing mTOR-interacting protein), and PRAS40 (proline-rich Akt substrate of 40 kDa). This complex can interact with the ULK complex (UNC-51-like kinase) composed of ULK1/2, Atg13 (autophagy related gene 13), FIP-200 (focal adhesion kinase family-interacting protein of 200 kDa) and Atg101. During nutrient-rich conditions, mTORC1 is activated and phosphorylates Atg13 and ULK1/2, which inhibits the ULK kinase activity thereby inhibiting the autophagy process. Conversely, when mTORC1 is inhibited, by starvation or rapamycin treatment for example, it dissociates from ULK complex, resulting in the activation of ULK and the induction of autophagosome formation.

The second macromolecular complex implicated in the initiation step of autophagy is the PI3K complex consisting of a class III PI3K, Vps34 (vacuolar protein sorting 34), p150, Beclin 1, and Atg14. This complex formation is necessary for Vsp34 activation to produce PI3P (phosphatidylinosositol-3-phosphate), a lipid molecule essential to the formation of autophagosomes through the recruitment of proteins required for the elongation of the phagophore [103,104]. Other regulatory proteins, including Ambra1, UVRAG (UV radiation resistance-associated tumor suppression gene protein), Rubicon, Bif-1 and the anti-apoptotic Bcl-2 or Bcl-xl can also interact with the PI3K complex to modulate its activity [105,106,107,108].

#### 3.1.2. Autophagosome Formation and Elongation

Once the autophagy is initiated, the phagophore elongates, leading to the formation of a double membrane vesicle, the autophagosome. Two ubiquitin-like conjugation systems are involved in the elongation of pre-autophagosomal structures. In the first conjugation system, Atg12 is conjugated to Atg5 by Atg7 (E1 ubiquitin-activating enzyme-like) and Atg10 (E2 ubiquitin-conjugase-like). Atg16L is then linked to Atg12-Atg5 via its coiled-coil domain to form a 800 kDa complex, which is essential for the elongation of pre-autophagosomal structures [109,110]. This macro-complex will dissociate from mature autophagosome.

The second conjugation system leads to the formation of LC3-PE (microtubule associated-protein 1 light chain 3-phosphatidylethanolamine) conjugate. The precursor of LC3 is cleaved at its COOH terminus by the protease Atg4B to generate the LC3-I form. LC3-I is then conjugated to PE by Atg7 (E1-like) and Atg3 (E2-like) to form LC3-II [111,112]. Unlike the Atg12-Atg5-Atg16L complex, LC3-II remains on completed autophagosomes until their fusion with lysosomes. At that time, LC3-II molecules present on the cytosolic face of the autophagosome will be recycled through cleavage by Atg4 [112] and LC3-II on the inner membrane of autophagosomes will be degraded within the autolysosome by proteolytic activity.

Connections between the two conjugation systems have been reported. It has been shown that the Atg12-Atg5 conjugate can have an E3 ubiquitin ligase activity to facilitate LC3 conjugation [113]. Moreover, it has been shown that the Atg12-Atg5-Atg16L complex and the interaction between Atg12 and Atg3 could specify the site of LC3 lipidation [114].

#### 3.1.3. Autophagosome/Lysosome Fusion and Degradation

In the final steps of autophagy, the mature autophagosomes fuse with lysosomes to form autolysosomes. In mammalian cells, the autophagosomes are formed randomly in the cytoplasm. Thus, autophagosomes need to reach lysosomes, mainly localized perinuclearly around the microtubule-organizing center (MTOC) [115]. This transport towards MTOC requires the involvement of microtubules and is mediated by the motor proteins dyneins [116,117]. Thus, inhibition of this transport, through depolymerization of microtubules or inhibition of motor proteins, could lead to an autophagy blockade [118].

Autophagosome-lysosome fusion is mediated by several proteins, including small GTPases (such as Rab7), syntaxin 5, several SNARES (soluble *N*-ethylmaleimide sensitive factor attachment protein receptor), LAMP1 and LAMP2 (lysosome-associated membrane protein) and the class C Vps proteins [119,120,121,122,123].

After fusion with the lysosome, the inner membrane of the autophagosome and the cytoplasmic material sequestered in autophagosomes are degraded by lysosomal acid hydrolases including proteinases A and B, and cathepsins B, D and L [124]. The resulting products of this degradation, especially amino acids, are then transported back to the cytosol for reuse.

It is interesting to note that, before the autophagosome/lysosome fusion, the autophagosome may also fuse with an endosome to form an amphisome, thus establishing a connection between endocytosis and autophagy [125]. Amphisome formation involves several proteins including Rab11, the HOPS complex, and components of multiple ESCRT (endosomal sorting complex required for transport) complexes that mediate cargo sorting into intraluminal vesicles of the multivesicular bodies [126,127].

#### 3.1.4. Evaluation of Autophagic Activity

Because autophagy is a dynamic process, there is an essential need to measure the autophagic flux; the assessment of the number of autophagosomes alone (by electron transmission microscopy or by quantifying the expression of LC3 protein expression) is indeed not enough to conclude about the efficiency of autophagy. LC3-II is associated with the autophagosome membrane, which makes it a useful marker of autophagosomes but not of autophagic flux. Indeed, the quantification of LC3-II protein expression at a given time is the result of a balance between LC3-II formation and degradation. Therefore, an analysis only based on LC3-II levels quantification cannot discriminate between two opposite scenarios: an autophagy activation (LC3-II formation) *versus* a blockade in the downstream step in autophagy (absence of LC3-II degradation), which implies a defective autophagy [128,129]. It is therefore essential, to be able to conclude about autophagy characterization and distinguish between these two possibilities autophagy pathway, to combine the analysis of autophagosome quantification with autophagic flux assays. Autophagy flux can be monitored using inhibitors of the fusion of autophagosomes with lysosomes. Lysosomotropic reagents such as bafilomycin A1 or chloroquine, which inhibit acidification inside lysosomes or the autophagosome-lysosome fusion, block LC3-II degradation leading to its accumulation. As a result, the difference of LC3-II levels between samples in the presence and in the absence of inhibitors reflects the autophagic flux. Similarly, inhibitors of lysosomal proteases such as E64d or pepstatin A can be used to measure autophagic flux [130]. Finally, autophagic flux can also be assessed by measuring the levels of substrates normally degraded by autophagy such as p62/SQSTM1 (sequestosome 1); p62 is a protein directly bound to LC3-II which acts as a cargo receptor for the degradation of ubiquitinated proteins targeted by autophagy. Increased levels of p62 are therefore reliable indicators of dysfunctional autophagy and increased autophagic flux is indicated by decreased p62 levels [131]. A detailed review on the methods for monitoring autophagy has been written recently by Klionsky *et al.* [128].

### 3.2. Autophagy in Physiological and Pathological Conditions

Basal autophagy, by maintaining cellular homeostasis, represents an important process in the physiology of many organs. A variety of functions, especially the elimination of organelles, macromolecules and pathogens, that are being assigned to autophagy can explain, at least in part, its involvement in a large number of diseases [96,97,132]. Autophagy dysfunction has been described in pulmonary diseases as in others organs. This will be discussed in the following paragraphs.

#### 3.2.1. Autophagy and Lung Diseases

Chronic obstructive pulmonary disease (COPD), which mainly results from chronic exposure to cigarette smoke, is the better studied lung disorder focusing on the role of autophagy. An accumulation of autophagosomes and enhanced levels of LC3-II and others autophagy-related proteins (Atg4, Atg5/12 and Atg7) have been observed in lung biopsies from patients with COPD [133]. An induction of autophagosome formation was also observed in different human lung cell types (epithelial cells and fibroblasts) exposed to cigarette smoke extract [133,134,135]. Furthermore, the genetic depletion of LC3-II or Egr-1 (early growth response-1), a molecule involved in LC3B transcription, was associated with a resistance to emphysema in mice after exposure to cigarette smoke [133,136]. These results suggested that the stimulation of the autophagic pathway might be deleterious, mainly by promoting airspace enlargement in response to cigarette smoke. Monick and colleagues also showed an accumulation of autophagosomes (TEM visualization and LC3-II level) as well as in alveolar macrophages from actively smoking patients, but also in alveolar macrophages of nonsmokers exposed to cigarette smoke extract *in vitro* [137]. Interestingly, by investigating the autophagic flux (using inhibitors studies and quantifying p62 expression), the authors concluded that the autophagy was not functional. They suggested that this autophagy defect leads to functional abnormalities of alveolar macrophages, especially an inability to clear bacteria from the alveolar surface (with a decreased lysosome delivery of bacteria in these cells), which could explain the recurrent infections observed in smokers such as in pneumonia. Taken together, these studies suggest an important role of autophagy in COPD progression.

Cystic fibrosis (CF) is a genetic disorder characterized by a mutation in the gene encoding the cystic fibrosis transmembrane conductance regulator (CFTR). In 2010, Luciani *et al.* showed that human and mice CF airway epithelia displayed defective basal autophagy as evidenced by decreased levels of LC3-II protein and increased levels of p62 [138]. Moreover, it has been observed that the defective CFTR protein leads to an autophagy inhibition by Beclin1 sequestration in aggresomes in airway epithelial cells, and that the restoration of autophagy by overexpression of Beclin1 restored CFTR trafficking, reduced its accumulation in aggresomes and reverts the CF airway phenotype [138]. Furthermore, treatment with rapamycin, an autophagy inducer, can limit the infection by *Burkholderiacenocepacia*, potentially lethal to CF patients, and decrease the associated inflammation in the lungs of CF mice [139]. Taken together, these studies suggest that autophagy deficiency is associated with cystic fibrosis and the related pulmonary inflammation.

Autophagy also seems to be implicated in the development of pulmonary arterial hypertension (PH). Indeed, elevated levels of LC3B were observed in an experimental mouse model of chronic hypoxia-induced PH but also in the lung of PH patients [140]. In addition to these data, it has been observed that LC3B^−/−^ mice, but also Egr1^−/−^ mice, showed an increased susceptibility to hypoxia induced-PH as compared to wild-type mice [140]. These results suggest that the autophagy pathway exerts a protective role during the pathogenesis of PH.

Similar effects of autophagy have been observed in others pulmonary diseases such as α1-antitrypsin deficiency, mycobacterium tuberculosis, tuberous sclerosis or acute lung injury and are discussed elsewhere [141,142,143].

#### 3.2.2. Autophagy and Cancer

The link between autophagy and cancer is complex given that autophagy can act both as tumor suppressor and tumor promoter. Indeed, on one hand, autophagy, which is able to degrade oncogenic proteins substrates, potential carcinogenic misfolded proteins and damaged organelles, can suppresses the initiation and the development of tumors. However, on the other hand, autophagy can assist the survival of established tumors by providing nutrients to tumoral cells that are in conditions of hypoxia and nutrient deprivation. Part of an explanation resides in the fact that many of the signaling pathways regulating autophagy imbricate with those regulating tumorigenesis. Indeed, several tumor suppressor genes such as PTEN, TSC1/2, UVRAG and p53 positively regulate autophagy [96,144]. Conversely, oncogenes such as class I PI3K, Akt or Bcl2 inhibit autophagy [107]. An example of the existing link between the autophagy machinery and human cancer has been proposed in a study by Liang *et al.* [145] published in 1999. The authors showed that, in a high percentage of human cancers (breast, ovarian and prostate cancers), Beclin1 gene was mono-allelically deleted and that its expression was decreased. Moreover, haploid-insufficiency of Beclin1 promotes tumorigenesis in various tissues in transgenic mice [146]. Similarly, homozygote deletion of Atg5 was shown to predispose mice to liver tumors [147]. Conversely, in some cases, autophagy can also promote the survival of cancers cells in inhospitable environments, when insufficient vascularization limits nutrient and oxygen supply to the cells [148,149]. Thus, induction of autophagy allows cancer cells to survive and subsequently favors tumor progression. Altogether, targeting autophagy in cancers should provide new opportunities for cancer treatment but these strategies could be complex to implement because of the dual role of autophagy in cancer.

#### 3.2.3. Autophagy, Inflammation and Oxidative Stress

Two common mechanisms might account for the important role of autophagy in physiological and pathological conditions; the interplay of autophagy with inflammation and oxidative stress.

Autophagy plays a crucial role in inflammation. Inflammasomes are multiproteins complexes which promote the processing and the secretion of the pro-inflammatory cytokines IL-1β and IL-18 [150]. Basal autophagy, by degrading cells debris or defective organelles which can activates the inflammasome, can negatively regulate inflammasome activation [151,152]. For example, an autophagy blockade leads to the accumulation of damaged mitochondria, which produce ROS, which in turn can activate the NLRP3 inflammasome [152]. In addition to control the production of cytokines by regulating the activation of inflammasomes, autophagy can also directly target pro-IL-1β for lysosomal degradation [153]. Importantly, Shi and colleagues, showed that the activation of inflammasomes in macrophages leads to the formation of autophagosomes and that a blockade of autophagy exacerbated the inflammasome activation [154]. These results indicate that there is a negative feedback loop where the inflammasome activation leads to an activation of autophagy that in turn negatively controls inflammation by clearing the active inflammasome. Moreover, efficient autophagy also limits inflammation by degrading apoptotic bodies, which can release damage-associated molecular pattern molecules, which can trigger inflammation [155].

In addition to these data, it has been shown that the endotoxin-induced production of IL-1β and IL-18 was enhanced in mice deficient for Atg16L1 [156]. Similarly, mice deficient for LC3b were found to be more susceptible to lipopolysaccharide than wild type mice, with higher serum concentrations of IL-1β and IL-18 [151]. Importantly, autophagy is also involved in the transcriptional regulation of genes involved in the inflammatory response. Indeed, when autophagy is deficient, p62 protein, a substrate of autophagy, accumulates and leads to TRAF6 (tumor necrosis factor receptor-associated factor 6) oligomerization and to the further activation of NF-kB, a transcription factor involved in inflammation [157,158,159]. Furthermore, autophagy genes are associated with inflammatory disorders such as Crohn’s disease, a chronic inflammatory disease of the intestine. Indeed, polymorphisms in autophagy-associated genes, such as Atg16L1, Irgm1 (immune-related GTPase M-1) but also Ulk1, are associated with Crohn’s disease [160,161].

The interplay between autophagy and oxidative stress occurs at the level of ROS production. Indeed, it is known that starvation can increase ROS production, in particular O_2_^−^ and H_2_O_2_, and thus activate autophagy [162,163]. In these studies, it has been shown that H_2_O_2_ induces autophagy via an inactivation of Atg4 at the site of autophagosomes formation, thus allowing the conversion of LC3-I to LC3-II, which, as mentioned before, is crucial for the initial steps of autophagy [163]. Moreover, autophagy has been shown to be induced in glioma cells treated with exogenous H_2_O_2_ through the Beclin 1 and Akt/mTOR pathways [164]. Further support for a role of ROS in the induction of autophagy comes from studies showing that the induction of autophagy by starvation is dependent of O_2_^−^ production, as the overexpression of SOD and/or catalase protects HeLa cells for starvation-induced autophagy [162].

Conversely, autophagy can also suppress ROS production. Indeed, impairment in the autophagy process leads to increased oxidative stress [165,166,167,168,169]. Moreover, autophagy plays a crucial role for the degradation of damaged mitochondria, which are the main sources of ROS generation. Indeed, a selective mitochondrial autophagy, called mitophagy, can act as a defense mechanism against oxidative stress by clearing damaged mitochondria [170]. For example, it has been shown, in mitophagy-deficient cells, that there was an increase production of ROS [171]. Additionally, several studies suggest that autophagy may have a key role in the degradation of oxidized proteins, in particular via the chaperone-mediated autophagy [172,173]. In response to oxidative stress, the nuclear factor (erythroid-derived 2)-like 2 transcription factor (Nrf2), involved in the transcription of antioxidants genes such as hemeoxygenase, can induce p62 expression which in turn, activates Nrf2, subsequently forming a positive feedback loop [174,175] to reduce the oxidative response. Moreover, because of its role in delivering protein aggregates to autophagosomes, p62 may be involved in the elimination of oxidized proteins.

**Figure 2:**
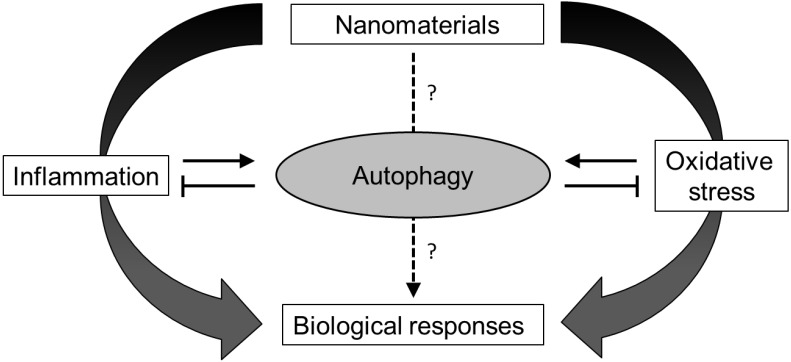
Hypothetic relationship between the autophagy and the biological responses to nanomaterial.

Overall, autophagy is a well-conserved physiological process aimed to maintain cellular homeostasis. Alterations of the autophagic process (*i.e*., abnormal activation and/or deficient activity) are associated with various diseases, and are probably link to the interplay between autophagy and inflammation and/or oxidative stress. Since these two mechanisms are largely described as underlying nanomaterials effects, the involvement of autophagy in nanomaterials toxicity has received a significant amount of interest lately and will be developed in the following paragraphs (Figure 2).

## 4. Nanomaterial-Induced Autophagy Perturbation

In this chapter, we chose to extend our review beyond the pulmonary perspective, to get a better overview of nanomaterial-induced autophagy perturbation.

### 4.1. Evidences of Autophagy Perturbation by Nanomaterials

When deciphering the literature on the subject, it appears that it is (roughly) divided in two major types of studies based on their experimental set-up; those limited to the observation of autophagosome (reported in Table 1), and those which go further and report analysis of the autophagic flux (reported in Table 2). As stated earlier, to be able to conclude on autophagy involvement in response to whatever exposure, it is essential to combine the analysis of autophagosome quantification with autophagic flux assays. However, given the important number of studies presenting a lack of deep investigation, and the incomplete associated conclusions, we chose to present the existing literature, as is it as of today.

Several classes of nanomaterials have been studied, mainly metal oxides NP and CNT. The large majority of studies used cell lines, a smaller amount using primary cells. Only rare studies showed data on animal models [176], probably because of the difficulty to use relevant methods to assess autophagy *in vivo*. A large part of the experiments reported increased numbers of autophagosomes in response to nanomaterials, observed either by the quantification of autophagosome markers expression (LC3-II, Atg5, *etc.*), or by TEM observations. This is true for metal oxides NP [177,178], graphene nanosheets [179] or silver nanowires [180] (Table 1). All these data suggest that nanomaterials can modify the autophagic pathway, essentially leading to the accumulation of autophagosomes. Some studies showed an implication of the Akt/mTOR pathway in these effects, such as that by Roy and co-workers showing an enhancement of autophagosome formation in mouse peritoneal macrophages exposed to zinc oxide NP, through the inhibition of the Akt/mTOR pathway, ultimately leading to apoptosis [181].

Going into deeper analysis, studies reported in Table 2 demonstrate that nanomaterials such as metal oxides [182,183], dendrimers [176] or CNT [184,185] can lead to an increased number of autophagosomes because of a blockade of autophagic flux. Indeed, Orecna and colleagues reported in HUVEC cells exposed to carboxylated MWCNT, an increased expression of LC3-II protein, without a further enhancement in presence of Bafilomycin A1, together with an increase of p62 protein expression [184]. In this case, the accumulation of autophagosomes can be attributed to a blockage in the autophagy flux; the autophagosomes accumulate, attested by an increase of LC3 levels, without the formation of autolysosomes (no degradation of p62 protein). Similar results have been reported for other nanomaterials such as SWCNT, graphene oxide or gold NP [185]. Interestingly, Sun and colleagues reported the only study demonstrating an increased autophagosome formation along with an increase of autophagy flux in A549 lung epithelial cells exposed to copper oxide NP [186].

**Table 1:** A summary of nanomaterial-induced autophagy dysfunction in the literature: focus on autophagosomes formation.

Nanomaterial	Model(s)	Autophagy markers	Experimental techniques	Results	Reference
Gold NP	MRC5 human lung fibroblast cell line	Beclin1, Atg5, Atg7, Atg12, LC3	TEM, immunoblot	Increase of autophagosomes formation	[187]
Iron oxide NP	RAW 264.7 murine peritoneal macrophage cell line	Beclin1, Atg5, LC3, p62	TEM, immunoblot, p62 immunostaining	Increase of autophagosomes formation	[178]
Silica NP	A549 lung epithelial cell line	LC3	TEM, MDC staining, immunoblot	Increase of autophagosomes formation	[188]
Silver NP	NIH 3T3 mouse embryonic fibroblasts	Beclin1, LC3, p62	TEM, acridin orange staining, immunoblot	Increase of autophagosomes formation	[177]
Zinc oxide NP	Mouse peritoneal macrophages	Atg5, Atg10, Atg12, LC3	TEM, qRT-PCR, LC3 immunostaining, immunoblot	Increase of autophagosomes formation	[181]
Hydroxyl C60 fullerene NP	HUVEC human umbilical vein endothelial cell line	LC3	TEM, immunoblot	Increase of autophagosomes formation	[189]
Polymeric NP	NR8383 rat alveolar macrophage cell line	Atg16L1, LC3	TEM, microarray, immunoblot, qRT-PCR	Increase of autophagosomes formation	[190]
Graphene oxide nanosheets	RAW 264.7 murine peritoneal macrophage cell line	Beclin1, LC3	TEM, immunoblot, immunostaining	Increase of autophagosomes formation	[179]
Silver nanowires	THP-1 monocytic cell line, iBMM cell line	LC3	TEM, stable GFP-LC3 transfection, immunoblot	Increase of autophagosomes formation	[180]

NP: nanoparticle, MDC: monodansylcadaverine.

In view of the current literature, it is currently not possible to definitively conclude on the interplay between nanomaterials and autophagy. Unfortunately, part of this situation is the result of inadequate methodological assessment of autophagy, too many studies only quantifying the number of autophagosomes to address the whole autophagy machinery. Excellent guidelines have been published recently in the literature and it is an absolute necessity to follow them to adequately conclude on nanomaterials-induced autophagy perturbations [128].

**Table 2:** A summary of nanomaterial-induced autophagy dysfunction in the literature: focus on modifications of autophagic flux.

Nanomaterial	Model(s)	Autophagy markers	Experimental techniques	Results	Reference
Copper oxide NP	A549 lung epithelial cell line	Atg5, LC3	TEM, immunoblot, GFP-LC3 transfection, Atg5 siRNA	Increase of autophagosome formation with an increase of autophagy flux	[186]
Iron oxide NP	A549 lung epithelial cell line	Akt signaling, Atg5, Atg12, LC3	Immunoblot	Accumulation of autophagosomes due to a decrease in autophagy flux	[182]
PAMAMdendrimer	A549 lung epithelial cell line, Balb/c mice	Atg6, LC3	TEM, immunoblot, GFP-LC3 transfection	Accumulation of autophagosomes due to a decrease in autophagy flux	[176]
MWCNT	A549 lung epithelial cell line	LC3	Immunoblot, qRT-PCR, GFP-LC3 transfection	Accumulation of autophagosomes due to a decrease in autophagy flux	[191]
SWCNT and graphene oxides	Mouse peritoneal macrophages	LC3, p62	GFP-LC3 transfection, immunoblot, lysotracker	Accumulation of autophagosomes due to a decrease in autophagy flux and lysosomal impairment	[185]
Carboxylated MWCNT	HUVEC human umbilical vein endothelial cell line	LC3, p62	TEM, immunoblot, RFP-LC3 and GFP-LC3 transfection	Accumulation of autophagosomes due to a decrease in autophagy flux	[184]

### 4.2. Mechanisms of Autophagy Perturbation by Nanomaterials

The mechanisms of nanomaterials-induced autophagy perturbation are not completely understood yet, but an impairment of the autophagosome-lysosome fusion and/or a defect in lysosome function could represent potential targets.

As stated before, the cytoskeleton, a highly dynamic cellular scaffold that supports cell shape and regulates the intracellular trafficking has an important role in autophagy. Indeed, several studies revealed the importance of the microtubular network, and to a lesser extent, of the actin cytoskeleton in the formation and the fusion of autophagosomes with lysosomes [192]. In rat hepatocytes and cells, the disruption of microtubules or actin microfilaments by agents such as nocodazole, vinblastine or cytochalasin B and D resulted in the accumulation of autophagic vacuoles, reflecting an inhibition of autophagic flux [193,194]. More recently, studies showed that, once formed, autophagosomes move along microtubules to concentrate at the perinuclear region around the MTOC, where the majority of the lysosomes are found, to fuse with them [115,195]. Moreover, in basal autophagy but not in starvation-induced autophagy, histone deacetylase-6 has been shown to control the fusion of autophagosomes to lysosomes by the actin remodelling machinery [196].

After passing the cell membrane, nanomaterials could interact with the proteins of the cytoskeleton, affect their functions and then, as described above, potentially lead to an impairment of the autophagy process. This has been particularly described for actin and tubulin proteins. For example, inhibition of tubulin polymerization by gold NP has been shown in a cell free system [197]. Moreover, fullerene derivative carbon NP and TiO_2_ NP were found to inhibit microtubule polymerization, potentially by a hydrogen bond between NP and the tubulin heterodimer [198,199]. More recently, a study described that SWCNT can directly bind to actin via hydrophobic interactions which leads to changes in actin structure [200]. A study on the effects of silicon dioxide NP on A549 cells reported differences in the expression levels of proteins associated with the regulation of actin cytoskeleton [201]. Gold nanomaterials have been shown to have a dose-dependent effect on actin stress fibers in human dermal fibroblasts, thereby inducing cytotoxicity [202]. Furthermore, in the same cell type, gold NP have been described to induce a disruption of the cytoskeleton, despite no change in actin and β-tubulin protein expression [203]. However, the disruption of the cytoskeleton was reversible given that the cytoskeleton could reconstitute following NP removal. Exposure to magnetic NP, such as iron oxide (Fe_2_O_3_) ones, could also alter cell function in pheocromocytoma neuronal cells by decreasing the number of actin filaments [204]. Consequently, these cells were not able to extend neurites in response to nerve growth factor. In PC12 cells, ferromagnetic mineral magnetite (Fe_3_O_4_) leads to alterations in microtubule polymerization, potentially induced by a direct bind to tubulin dimer [205]. Moreover, exposure to iron oxide nanomaterials on human umbilical vein endothelial cells leads to a significant disruption of cytoskeletal structures, with diminished vinculin spots, and disorganized actin and tubulin networks. Interestingly, in addition to the observed cytoskeleton disruption, this study also suggests an autophagy dysfunction that could explain the toxic effects of particles [206]. Similar effects on cytoskeleton were observed in response to a high concentration of various Fe_2_O_3_ nanomaterials in murine neural progenitor cells and human blood outgrowth endothelial cells [207]. Additionally, researchers have shown that fullerenol treated renal proximal tubule cells displayed actin disruption and clamping associated with autophagic vacuole accumulation. The authors suggest that cytoskeleton disruption, by interfering with the autophagy processing, may be an explaining mechanism of fullerenol cytotoxicity [208].

Because of the involvement of lysosomes in the final steps of the autophagy process, a lysosome dysfunction could also be a mechanism explaining a defect of the autophagy pathway leading to nanomaterials-induced toxicity. Indeed, several types of nanomaterials have been recognized as being associated to lysosomal dysfunction. For example, MWCNT, with a diameter <8 nm, induced lysosomal membrane destabilization (LMD) in 3T3 fibroblasts, leading to the release of lysosomal contents inside the cytoplasm. This was associated with an increased ROS production [209]. However, no or minor lysosomal damage were observed with larger MWCNT, or with nanomaterials of different composition (TiO_2_, SiO_2_) and in other cell types (telomerase-immortalized human bronchiolar epithelial cells and RAW 264.7 macrophages). In another study, G5-polyamidoamine dendrimers have been shown to be taken up into the lysosomal compartment and to modify the lysosomal pH, increasing the cytotoxicity [210]. Likewise, gold NP can accumulate in lysosomes and cause lysosomal dysfunction by increasing the lysosomal pH in rat kidney cells [183]. Interestingly, a blockade of the autophagy flux was also observed in these cells, suggesting lysosomal dysfunction as a likely mechanism of autophagy blockade. However, although most studies showed an elevation of the lysosomal pH in response to treatment with NP, this propensity of NP to accumulate in lysosomes could be presented as a strategy to treat lysosomal defects. Indeed, researchers used acidic NP to lower the pH of compromised lysosomes in human retinal pigment epithelial cells and thus to improve their degradative function [211]. The occurrence of lysosomal destabilization as a mechanism of TiO_2_ NP-induced cytotoxicity has also been proposed in mouse fibroblast cells and in bronchial epithelial cells [212,213]. Moreover, it has been shown that exposure to zinc oxide NP in human monocytic THP-1 cells induced a decrease of lysosomal stability together with a loss of viability [214]. The authors suggest that the lysosomes were destabilized by the production of Zn^2+^ ions formed by the dissolution of ZnO NP into the acidic lysosomes. The release of the lysosomal content and Zn^2+^ ions into the cytoplasm may damage other organelles and lead to cell death. Likewise, TiO_2_ nanobelts and amino-functionalized polysterene NP have been shown to induce toxicity by a loss of lysosomal integrity and a subsequent release of cathepsins which could lead to cell death, oxidative stress and inflammation [215,216]. Recently, in mouse peritoneal macrophages, CNT have been shown to induce lysosome impairment, characterized by an overload of lysosomes by nanomaterials and a decreased lysosomal stability and biogenesis, associated with a dysfunction of autophagy [185].

**Figure 3:**
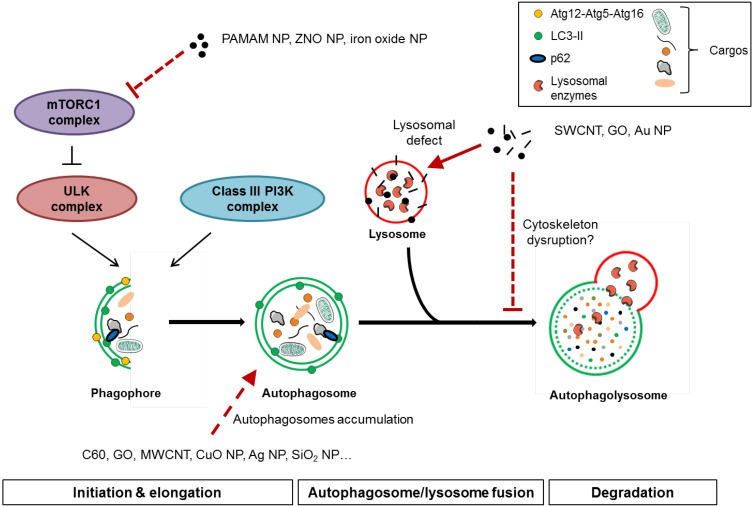
Nanomaterial-induced autophagy perturbation. Full lines relate to direct evidences of interaction of nanoparticles (NP) with the autophagic process whereas dotted lines relate indirect evidences of such interactions.

Taken together, the disruption of cytoskeleton together with a defect in the lysosome function could represent essential mechanisms explaining how nanomaterials could perturbate the autophagy process (see Figure 3). However, these mechanisms deserve further attention, as they are far from being completely understood yet.

## 5. Conclusions

In conclusion, autophagy dysfunction could represent a good candidate to explain, at least in part, nanomaterials toxicity. The deep understanding of the underlying mechanisms involved in this interaction between nanomaterials and the autophagy process certainly deserve much attention from researchers in the nanotoxicity-field to help the development of safer nanomaterials.
